# Anæsthesia

**Published:** 1887-02

**Authors:** F. F. Lord


### Anæsthesia; The Administration, and Mode of Action of Anæsthetics.

BY F. F. LORD, M. D.

(Read before the California State Dental Association.)

*Mr. President and Gentlemen:*

Absence of feeling is death in the degree of its presence.

Though many agents may be denominated anæsthetics, a word taken from the Greek which means, "an agent or agents that produce insensibility or prevent feeling," yet I will only describe the principal agents that are used at the present time to produce anææsthesia, viz: the terchloride of formyl, or as it is most commonly called chloroform; and oxide of ethyl or ether, commonly and improperly called sulphuric ether. The discoverers of anæsthetics ought to be remembered with everlasting gratitude by the whole civilized world, the names of Samuel Guihae, Dr. Horace Wells, and Dr. Morton, ought to be engraved in every one's memory.

In 1844 Horace Wells, a dentist, after the first success obtained by experimenting on himself with nitrous oxide, was so hissed by the students on making his first public experiment, that he gave up the practice of dentistry and kept a menagerie. Since that a statue has been raised to him. Let us look at the past life of Dr. Wm. T. G. Morton, who was a pupil of Wells. Instead of using nitrous oxide, he, in 1846, used the vapor of ether for extracting teeth. Dr. Morton may justly be called the inventor and revealer of anæsthesia. Having exhausted his means in advancing anæsthesia, and becoming reduced in circumstances, he brought suit against the Government for infringement of his patent, but without success. Contributions were solicited to relieve him, but from some unknown and unaccountable cause, the efforts failed ; as did also a suit against the various hospitals and infirmaries in which ether had been employed. Dr. Morton finally died a discouraged, disheartened, and penniless man. His remains rest in Mount Auburn Cemetery, near Boston, without a stone to mark the spot. What a homily could be read on this history! What a lesson does it teach us! I can only say with gratitude of Dr. Morton, that he was the one who robbed the surgeon's knife of half its terror, and this day perhaps, some wounded veteran beholds for the last time his mutilated members, but with unlimited confidence "wraps the drapery of his couch about him, and lies down to pleasant dreams." While therefore, we remember with gratitude the originator, may we not, as a profession, with just pride congratulate ourselves that in using anæsthetics, we have so great a boon for suffering humanity?

Before administering these agents, the patient ought to undergo an examination by the medical man, in regard to his health, and if he is troubled with any of the following diseases, it ought not to be administered; still if it must be given, give it very cautiously. For instance, in heart disease, especially where the muscular substance has undergone fatty degeneration ; in mitral disease, in hypertrophy, and in dilated aorta, or in cases of aneurism of the principal arteries given off from the aorta ; in asthma, hydrothorax, epilepsy, idiocy, paralysis, and acute mania, and in the later stages of phthisis. In bronchial affections, the vapor is apt to produce troublesome cough, and in hysterical patients, these preparations may cause laryngeal spasm ; in renal disease, where the blood is loaded with urea, it is dangerous. Hard drinkers should not be anæsthetized with chloroform, for to them it is more than ordinarily dangerous. In active congestion or acute inflammation of the stomach, lungs, or brain, or where there is a tendency to hemorrhagic diathesis, or a generally plethoric condition. In some of these diseases any shock such as the extraction of a tooth is attended with danger, either with or without an anæsthetic. It is imperative that the surgeon should have confidence in the agent, and an intimate acquaintance with its various properties. He should have judgment sufficient to discriminate as to when and to whom it should be administered. After he has made an examination, he must loosen every article of clothing around the neck, chest, and waist, and place the patient in a recumbent position. He must also see that the patient has fasted three or four hours before the administration of the anæsthetic begins, and no accumulation must be suffered to take place in the bowels, more especially in cases of obesity. One rule is laid down most stringently by the great master of anæsthetics, and is the one which is most studiously avoided, especially in the hospital practice. I refer to Simpson's rule, that absolute silence should be maintained in the room during the administration of the anæsthetic. There should be no talking, no walking, no slamming of doors, until complete unconsciousness has occurred. Not a sound should be heard in the room, save the gently repeated request of the administrator to the patient, to keep the eyes shut and take long breaths. Instead of this, how often do we hear garrulous bystanders, loquacious operators, or officious nurses suggesting to the patient, in the last moments of his consciousness, ideas which often run into action and produce the excitement and struggling, for which the innocent anæsthetic is most wrongfully blamed. No one can appreciate the importance of absolute silence on the part of the bystanders during the administration of an anæsthetic, unless he has himself had an anæsthetic administered to him.

The mind should be free from all fear, either in regard to the possibility of pain or dangerous consequences from the inhalation; not that the imagination of evil effect will cause dangerous consequences, but it may make a very unpleasant and disagreeable operation, of what otherwise would be a delightful one to the surgeon. There should be a perfect willingness or even anxiety to inhale it, and the thoughts should be fixed as much as possible upon some pleasing subject, such as music, scenery, travel, etc. In ninety per cent. of cases where persons fail to become perfectly unconscious or where they express themselves as being disagreeably affected, the cause is to be found in some dread or fear that impresses itself firmly upon the mind at the outset.

One common cause, especially with ladies, is the determination while anæsthetized, not to say anything that may cause them embarrassment. Such anxiety, I believe to be perfectly unjustifiable. Death frequently occurs from surgeons being too hasty in putting patients under the influence of the anæsthetic by pouring too large a quantity of chloroform on the towel to control the struggles of the patient before the operation. Chloroform should be poured in small quantities on the towel which is simply folded, then brought gradually to the face and held within half an inch of the patient's nose, never allowing it to touch, especially when a fresh supply of chloroform has been poured on the towel. Free admission of air with the anæsthetic is necessary; three and a half per cent. being the average amount, and four and a half the maximum proportions of chloroform to atmospheric air.

Chloroform should be given very slowly so that it will take at least fifteen minutes to prepare the patient for the operation. Never give it fast enough to sensibly diminish the rythm or volume of the pulse, or to darken the color of the surface, and after the condition of insensibility is reached, take the towel away and only apply it again when the patient is partially recovering. Then give it again slowly and by taking proper care, I think that operations may be safely performed on very small children and on old persons.

With those whose lungs or hearts are diseased or who are much reduced by chronic diseases of various kinds, I think that the best mode is to give the patient a little stimulant, such as brandy or ammonia before commencing the operation; then use ether, and when the patient is almost under the influence of the anæsthetic, give a few whifs of chloroform so that when you operate he is fully under the influence of the anæsthetic. I give the stimulant for rousing the action of the vascular and respiratory systems, especially to guard against cardiac syncope, I give the ether also as a tonic to the heart's action, then I give the patient the chloroform, as he will remain longer under its influence. Nearly all deaths occur in the first stages of chloroformization, and in using ether, first we use a stimulant to the heart's action, which is just the reverse in using chloroform. In chloroform we find twelve parts of carbon to one of hydrogen, the balance, one hundred and five parts, is chlorine. Chloroform is not therefore an acid or an alkali, but applied locally it first acts as a diffusible stimulant, then as an escharotic; just so does it act generally. Consequently it has a tendency to abolish sympathetic function and paralyze the heart.

Ninety per cent. of all the deaths from its administration occur during the beginning of its exhibition, and what seems more strange is that these subjects are from the healthy class, instead of the cachectic.

Our profession can reduce this mortality greatly, because of the fact that a large proportion of these deaths is directly traceable to one of four causes, to-wit: the position of the body, impurity of the chloroform, mental shock, or asphyxia, which also encourages us to hope that this margin may be greatly reduced, for I believe that all of these four prolific causes of death can be removed.

Asphyxia is caused by too great exclusion of atmospheric air, differing from syncope, since this is occasioned by an overdose, the chloroform acting subsequent to its removal from the patient, having accumulated in different parts of the body (for which it has a great affinity), transforming the blood, and producing formic acid ultimately. A remedy, therefore, for asphyxia is to dilute the vapor by the use of mechanical means, giving a very low percentage of it at first. At a temperature of 60° the atmosphere will take up this vapor about 12 per cent. According to the best authorities six per cent. is the utmost limit of safety. (This even is fatal to brutes.) Chloroform should be given on a towel, held at first a few inches from the face; and the first few inhalations should be taken with full inspirations. The towel is then brought gradually nearer the face until it is about half an inch away, and kept there. This gives about one per cent. at first, and four and a half per cent. when the towel is about half an inch from the face.

Mental shock occasioned by fear, concussion, or an effect of the will by muscular persons not to succumb, is a subject for unlimited skill to calm. The remedy for position and impurity is apparent. One strong influence is that this anæsthetic has an affinity for, and dissolves the haematin, upon which the color of the blood is supposed to depend, and that the anæsthetic commonly commences about the time it has made the circuit of the body. If, however, by diluting and regulating the quantity of vapor inhaled in a given time, this action can be kept so low as to prevent the production of formic acid, and we avoid all known causes of asphyxia, not forgetting to give it when the stomach is empty, we may then look to aneurism of the aorta, as the greatest of all counter-indications. The best time to operate is just after muscular tremor, or just after voluntary action ceases. Some of the signs of danger are syncope, pallor, prostration of the arteries, and the heart's action. Failure of the circulation is a primary sign of danger, and a pulse below forty, alarming; in which case no time should be lost, and no perseverence spared in resuscitation. Sylvester's or Halls's method should at once be called into use, and the galvanic battery used at the same time. Ordinary stertor or snoring, turgid vessels, and a red face indicate a regular pulse, but that stertor which more resembles the bray of a certain hybrid, is ominous of formic acid. Mixtures of ether, alcohol, etc., with chloroform, seems to be wrong in theory, and productive of little good. A mixture of one-half alcohol is by far the best, inasmuch as only about one-half as much chloroform can be evaporated in a given time, the mixture rendering it safer. As long as chloroform is used to produce anæsthesia it will continue to claim its occasional victims.

*Ether.* Why ether should be preferred and used instead of chloroform:

First. Ether stimulates the heart's action and uniformly improves the pulse, while chloroform paralyzes the heart.

Second. Ether, from its stimulating quality, is antagonistic to the effect of the shock of an operation.

Third. There is a greater liability to vomit before and after the operation, when chloroform is used, than with ether. Respiratory paralysis is produced by ether when circulation and blood pressure remain within the limits compatible with life. Sometimes the vascular pressure increases, sometimes it decreases; but it is always sufficiently high to allow the exchange of carbonic acid gas with the oxygen of the atmosphere. The cessation of respiration, therefore, is not the most dangerous moment to animal life when ether is employed.

It may be so with chloroform, because sometimes it is possible to produce some automatic respiratory movements, but nevertheless respiration ceases immediately, and the animal dies when chloroform is administered. With ether, on the contrary, when some automatic inspirations are obtained, one may be certain that respiration will continue, and that the animal will live. When chloroform is used and vascular paralysis occurs and lasts thirty seconds, artificial respiration is useless, because there is no longer any change of gases, the blood pressure being diminished. We see that vascular succeeds respiratory paralysis when ether is administered, whilst the reverse takes place with chloroform, so that with our present knowledge there are no means which will show us how to recognize, so as to prevent the tendencies which may cause death in some animals after the first inhalations of chloroform, before having produced true anæsthesia. The reverse occurs with ether; so that it may be said that in the present state of knowledge the surgeon is responsible for the death of an individual by etherization, whilst he is not responsible when death occurs during chloroformization. The following conclusions may now be formed:

First. The phenomena relating to the paralysis of sensibility and movement are common to both ether and chloroform.

Second. The two other phenomena, those relating to vascular and respiratory paralysis, often show themselves in inverse order with reference to these two agents.

Third. With chloroform, however, either the one or the other of these two paralyses may first show itself involving great danger to the individual if the vascular phenomena be the first to make their appearance. Ether should be administered on a towel folded in the shape of a cap or cone. Before folding the towel place a newspaper within the folds of the towel to prevent evaporation. Into this ether cap first place cotton and press it firmly to the bottom; upon this lay a good-sized sponge and the cap is ready for use. This cap is fitted as accurately as possible to the face, the towel accommodating itself to all inequalities of surface, which none of the patented inhalers can do. Acetic acid results but rarely from its careful handling. In conclusion if the position be tenable that so administering pure ether (with a slight admixture of air) that none of the vapor can escape into the room, thereby concentrating its power, then the number of that class of patients may be very much lessened, of whom it is said they cannot be brought under its influence, and neither the surgeon nor his assistants will be subject to its tenacious, and to some, intolerable odor, which, added to the now common nausea, forms so great an objection to its use. For the above reasons chloroform should be rejected, and ether only be used.

Death from Anæsthesia. This may be said to be a sort of necessary price paid for the advantages of anæsthesia. Death from chloroform may occur in three different ways, viz: by coma, by asphyxia, and by syncope—through the brain, through the lungs, and through the heart.

Death from coma, or through the brain, may occur in persons who have epileptic attacks, also persons who are suffering from chronic Bright's disease, especially those that have symptoms of uræmia. Death seems to result from the dark, venous blood circulating through the venous centres. Death from asphyxia, or through the lungs, may be caused by tight clothing or anything which hinders the respiratory movements, as a large abdominal tumor—by actual choking. Sufficient air should be admitted with the chloroform vapor to maintain the respiratory function, the proportion I have given when speaking on chloroform. When the peculiar laryngeal stertor is produced the an-

æsthetic should be discontinued. If chloroform be given too soon after a meal, vomiting of semi-digested food is very likely to occur, and the larynx may be obstructed by it. The tongue may become paralyzed when the patient is fully under the influence of the anæsthetic and fall back into the throat, causing choking. A set of false teeth, or a tooth after extraction may slip down into the throat and cause suffocation. This has happened quite often in dental practice and caused death.

Third. Death from cardiac syncope. This is the most dangerous cause. A patient with a weak or fatty heart is in a most dangerous, even critical state when the lungs are filled with chloroform vapor which cannot escape. This may be caused when the towel is held too near to the face or by some other mechanical obstruction, such as teeth falling into the trachea. The chloroform vapor becomes diffused and cannot escape, the pulmonary circulation is obstructed and the pressure on the right ventricle becomes greatly increased. There is a weak, propulsive power, the organ cannot recover itself, and the result is fatal.

Chloroform has always, especially at first, a slight asphyxiating tendency; the patient calls out that he is choking, tries to pull away the inhaler, and breathes deeply, then struggles and holds his breath for a few seconds. Now if the patient has a weak and enfeebled heart and the pressure caused by the chloroform vapor in the lungs is too great and he holds his breath, should a ventricular contraction be missed from this cause it is a most difficult thing to start again—generally all is over. Chloroform in some cases acts directly on the heart by paralyzing its action. The patient suddenly becomes pale, the pulse generally beats in a flickering manner a few moments and the patient is dead. An autopsy reveals the valves, as a rule, healthy, but a weak and fatty heart. The walls are generally thin and the muscular tissue pale and degenerated.

Resuscitation, after poisoning with anæsthetics is by artificial respiration. Marshall Hall's or Sylvester's method should be tried. This is by the alternate and steady compression and relaxation of the walls of the patient's chest, or the surgeon should apply his mouth to the patient's lips and thus breathe into the chest, so as to empty the lungs of the vapor contained in the air cells, and to aid the oxygenation of the blood. By this means resuscitation may sometimes be accomplished after natural respiration has ceased, provided the heart continues to act. It may sometimes be effected even after the cessation of the heart's action, but this result is exceptional.

Galvanism resuscitates within the same limits as artificial respiration. One pole, the positive, is placed to the neck in a line with the fourth cervical vertebra, to the phrenic nerve; and the other, the negative pole, over the diaphragm. You may also place one pole to the upper dorsal spinous processes and the other over the apex of the heart, or both poles to the fourth cervical vertebra, so as to stimulate the phrenic nerves and aid respiration and the heart's action. With either remedy it is found that animals quickly rendered insensible by a strong dose more easily recover than those which have been gradually narcotized, even by a small percentage of the anæsthetic. In case of accident in the more threatening conditions, artificial respiration is to be commenced instantly, and this equally in all cases, whether the respiration alone has failed or both the pulse and respiration. Galvanism may be used in addition; but artificial respiration is on no account to be delayed or suspended in order that galvanism may be applied. Half an ounce of brandy may be given per rectum and the lungs be inflated by means of a catheter.

An excellent method of restoring animation is the inhalation of pure oxygen gas. If it can be procured in season it may be of great service. You must cease the administratien of the anæsthetic at once, when the peculiar laryngeal stertor is produced; and if this passes on to complete obstruction of respiration the tongue must be forcibly pulled forward so as to cause a reaction of the arytenoid cartilage by reflex action, and not merely to bring the tip of the tongue just in front of the teeth, as is usually done, under the impression that the obstruction is due to the falling back of the tongue. Fresh air should be admitted by opening doors and windows, and all bystanders should be sent out of the room. Cold water should be dashed into the face of the patient and friction to the extremities may be tried. The chest may be smartly slapped with a towel dipped in ice water, and a piece of ice may be introduced into the rectum. The patient should be placed in a horizontal position, thus throwing a large volume of blood upon the brain. The nitrite of amyl has lately been tried and found very efficient. It is put up in pearls of glass, each containing five drops, which may be carried in the vest pocket. It should be broken with the fingers, in a handkerchief, and applied to the nostrils when needed. Acupuncture of the heart has lately been suggested, but its usefulness has not yet been determined. If practiced, it should only be as a last resort, when all other means have failed.

Dr. Lord.—By the way, I presume a majority of the members may recollect this case. I happened to come in right after the administration of the anæsthetic. A young man, twenty-eight years old, came in and wanted to have two teeth extracted. The dentist had not graduated at that time. He has returned to the city and made himself quite conspicuous again. I had an engagement with him that day to do something for me. Just as I came into the office he told me he had administered some ether and the patient felt very bad, and he asked me to go in and see him. I went into the room and looked at him. He told me he had quite a severe headache and felt very dizzy. I assisted him out of the chair and laid him on a sofa, and administered some aromatic spirits of ammonia. After that he felt a little better and I told him to go home. Soon after he became worse and called in his family physician, Dr. Miller. Two days later his brother came into my office and wanted me to go down to see him. I complied, and found him to be a raving maniac. There were three men holding him down. He had these men to attend him night and day. He lived five days afterwards. The autopsy revealed a rupture of a cerebral artery. Now, to say the truth, I think that this was really caused by the ether being in an impure state. This doctor had always been in the habit of using nitrous oxide. He had a gasometer and always prepared the gas himself, but he was going to the East that afternoon, and the gas had given out, so he administered ether. In my opinion, because I was pretty intimately acquainted with him at that time, he did not habitually give either, and the ether might have been on his operating table for a year or a year and a half, and instead of being ether it was merely acetic acid. I attribute the cause of his death more to the impurity of the ether than to anything else.

Since the last convention of this Association there has been another case similar to the last. A young lady with whom I was well acquainted, having known her ever since she was a child, went to a dentist on Market street. She had some teeth removed while under the influence of laughing gas. When she recovered consciousness she felt in the same condition as this gentleman. She was about 18 or 19 years old at the time this happened. She was taken home in a hack and became much worse. At the present time she is in our State Insane Asylum. Now, gentlemen, what I was going to say is this: I consider this more or less due to the impurity of the laughing gas. In our offices we usually have assistants. We do not make our own gas, but leave it, as a rule, to boys who have perhaps not been in our offices more than a few months. They are the ones who make our gas. I think if this dentist had made his own gas the accident might not have happened.

There is another case that I saw, in which death took place. That was at the County Hospital. A woman of about forty years of age was brought in and laid upon the operating table. She had necrosis of the lower jaw. In this case chloroform was administered. I don't think that she was under its influence more than four minutes, when the operating surgeon looked at her and found her to be dead. Of course all methods of resuscitation were tried but they were unavailing. Of course there are cases that happen in private practice, and in hospital practice, perhaps, that are not recorded. If a case occurs in hospital practice the surgeon generally claims that the party dies from exhaustion, or the shock of the operation, or no mention is made of it whatever. Of course in private practice we have to be a little more careful. These are several cases that came under my observation during the last year, regarding anæsthetics, where death occurred in two cases, and another case where the young lady is in the Stockton Insane Asylum.

I will state, Mr. President, before I sit down, that I was out at the pound some weeks since and performed some operations on dogs. If I can find the notes before the Convention adjourns I will report on them.

